# Bacterial DNA induces the formation of heat-resistant disease-associated proteins in human plasma

**DOI:** 10.1038/s41598-019-54618-9

**Published:** 2019-11-29

**Authors:** Victor Tetz, George Tetz

**Affiliations:** 1 Human Microbiology Institute, New York, NY 10027 USA; 2 Tetz Laboratories, New York, NY 10027 USA

**Keywords:** Cancer, Pathogens

## Abstract

Our study demonstrated for the first time that bacterial extracellular DNA (eDNA) can change the thermal behavior of specific human plasma proteins, leading to an elevation of the heat-resistant protein fraction, as well as to *de novo* acquisition of heat-resistance. In fact, the majority of these proteins were not known to be heat-resistant nor do they possess any prion-like domain. Proteins found to become heat-resistant following DNA exposure were named “Tetz-proteins”. Interestingly, plasma proteins that become heat-resistant following treatment with bacterial eDNA are known to be associated with cancer. In pancreatic cancer, the proportion of proteins exhibiting eDNA-induced changes in thermal behavior was found to be particularly elevated. Therefore, we analyzed the heat-resistant proteome in the plasma of healthy subjects and in patients with pancreatic cancer and found that exposure to bacterial eDNA made the proteome of healthy subjects more similar to that of cancer patients. These findings open a discussion on the possible novel role of eDNA in disease development following its interaction with specific proteins, including those involved in multifactorial diseases such as cancer.

## Introduction

The role of microbiota is being revisited due to its emerging role in pathologies that were previously considered non-microbial^1,2^. For instance, bacteriophages have been recently found to be associated with the development of specific human diseases, such as Parkinson’s disease and type 1 diabetes^3–5^. Moreover, particular attention has been paid to pathogen-associated molecular patterns (PAMPs), mainly represented by components of microbial biofilms, including those of the gut microbiota^6^. One example is bacterial extracellular DNA (eDNA). Bacteria produce large amounts of eDNA that plays a multifunctional role in microbial biofilms, as a structural component, a nutrient during starvation, a promoter of colony spreading, and a pool for horizontal gene transfer^7–9^. eDNA is also known to affect bacterial protein modification in biofilm matrix, as exemplified by its role in the conversion of bacterial water-soluble proteins into extracellular insoluble β-sheet-rich amyloid structures, such as self-propagation and resistance to proteases and heat^10–12^. Heat resistance is a hallmark of prion proteins, although its biological significance is not clear. Notably, heat resistance is not an exclusive property of prion proteins or proteins implicated in heat-shock events, but can also be due to the occurrence of specific mutations in mammalian proteins that are normally not thermo-resistant, which makes this phenomenon even more puzzling^13,14^.

The properties of bacterial eDNA have been poorly investigated, except for its actions in the context of microbial biofilms. On the other hand, the chances that the eDNA secreted from microbial communities interacts with human proteins are relatively high. For example, eDNA released during biofilm spreading or lytic bacteriophage infection can enter the systemic circulation by different pathways, also facilitated by the altered intestinal permeability that accompanies the increased absorption of PAMPs^15–17^. Increasing evidence shows that impaired gut barrier dysfunction is an important determinant for the increase in circulating bacterial DNA that is associated with different diseases. Indeed, increased levels of both bacterial eDNA and human cfDNA characterize various pathological human conditions including cancer, stroke, traumas, autoimmune disorders, and sepsis^18–21^.

Another way by which PAMPs can enter biological fluids is their release from bacteria localized within the “internal environment” such as brain or placenta^22–25^. Moreover, DNA can be released into eukaryotic cells from obligate and facultative intracellular bacteria^26,27^.

Thus, despite the fact that interactions between bacterial eDNA and humans are very likely to occur, the effects of bacterial eDNA within body fluids are poorly studied, except for the CpG motif-induced activation of proinflammatory reactions through Toll-like receptor 9^28^. In this study, we evaluated a novel effect of bacterial eDNA on blood plasma proteins, which resulted in the alteration of the heat resistance of these proteins.

## Results

### eDNA-induced alteration of protein heat resistance in the plasma of healthy controls

We first studied the effects of DNA on the thermal behavior of proteins from the plasma of healthy individuals. Most proteins were aggregated after boiling, and the supernatant contained heat-resistant fractions of over 100 proteins. The identified heat-resistant proteins had a molecular weight ranging between 8 kDa and 263 kDa. Treatment with bacterial and human buffy coat DNA altered the composition of the heat-resistant protein fraction. We first verified which plasma proteins, among those that were heat-resistant before treatment with DNA, exhibited an increased level following DNA exposure in at least one healthy control (Table 1).

**Table 1:** Heat-resistant proteins of healthy controls whose amount increased following treatment with different DNAs.

N	Accession No UniProt	Uniprot Accession	Protein name
**eDNA of** ***P. aeruginosa***			
1	P02768	ALBU_HUMAN	Serum albumin
2	P02751	FINC_HUMAN	Fibronectin
3	B4E1Z4	B4E1Z4_HUMA	cDNA FLJ55673, highly similar to Complement factor B
4	P02774	VTDB_HUMAN	Vitamin D-binding protein
5	P01859	IGHG2_HUMAN	Immunoglobulin heavy constant gamma 2
6	P00747	PLMN_HUMAN	Plasminogen
7	Q14624	ITIH4_HUMAN	Inter-alpha-trypsin inhibitor heavy chain H4
8	Q5T987	ITIH2_HUMAN	Inter-alpha-trypsin inhibitor heavy chain H2
9	P04114	APOB_HUMAN	Apolipoprotein B-100
10	O14791	APOL1_HUMAN	Apolipoprotein L1
11	P19652	A1AG2_HUMAN	Alpha-1-acid glycoprotein 2
12	P20851	C4BPB_HUMAN	C4b-binding protein beta chain
13	P01857	IGHG1_HUMAN	Immunoglobulin heavy constant gamma 1
**eDNA of S.aureus**			
1	P02652	APOA2_HUMAN	Apolipoprotein A-II
**eDNA of S.mitis**			
1	P02652	APOA2_HUMAN	Apolipoprotein A-II
**eDNA of E.coli**			
1	P19652	A1AG2_HUMAN	Alpha-1-acid glycoprotein 2
2	P04114	APOB_HUMAN	Apolipoprotein B-100
3	P20851	C4BPB_HUMAN	C4b-binding protein beta chain

^*^Significant fold change in the level of heat-resistant proteins between normal plasma and plasma treated with eDNA for the proteins with spectrum counts <200 and over 30% increase for the proteins with spectrum counts >200*.

We next measured the increase in heat-resistant protein fractions following the treatment of plasma with bacterial eDNA. The highest increase in heat-resistant fractions of different unrelated proteins was registered after incubation with the eDNA of *Pseudomonas aeruginosa*. Interestingly, eDNA from different bacteria produced distinct effects. Indeed, the exposure to eDNA from the gram-positive bacteria, *Staphylococcus aureus* and *Streptococcus mitis* resulted in a selective increase in heat-resistant APOA2, which was not observed after treatment with eDNA from gram-negative bacteria. Under the same conditions, *E. coli* eDNA increased the heat-resistant fractions of A1AG2, APOB, and C4BP; however, the latter heat-resistant fractions were also increased after exposure to *P. aeruginosa* eDNA.

Intriguingly, specific proteins that did not exhibit a heat-resistant fraction in untreated plasma samples became heat-resistant following eDNA exposure. Table 2 lists the proteins that displayed such a behavior in at least one of the plasma samples.

**Table 2:** Proteins that became heat-resistant following eDNA treatment but had no heat resistant fractions before.

N	Accession No UniProt	Uniprot Accession	Protein name
**eDNA of P.aeruginosa**			
1	P69905	HBA_HUMAN	Hemoglobin subunit alpha
2	Q03591	FHR1_HUMAN	Complement factor H-related protein 1
3	P01031	CO5_HUMAN	Complement C5
4	A0M8Q6	IGLC7_HUMAN	Immunoglobulin lambda constant 7
5	O43866	CD5L_HUMAN	CD5 antigen-like
6	P49908	SEPP1_HUMAN	Selenoprotein P
7	P0DOY3	IGLC3_HUMAN	Immunoglobulin lambda constant 3
8	P63241	IF5A1_HUMAN	Eukaryotic translation initiation factor 5A-1
9	P04264	K2C1_HUMAN	Cluster of Keratin, type II cytoskeletal 1
10	P35527	K1C9_HUMAN	Keratin, type I cytoskeletal 9
11	P13645	K1C10_HUMAN	Keratin, type I cytoskeletal 10
12	A0A075B6S5	KV127_HUMAN	Immunoglobulin kappa variable 1–27
**eDNA of** E.coli			
1	Q9P2D1	CHD7_HUMAN	Chromodomain-helicase-DNA-binding protein 7
2	Q9UGM5	FETUB_HUMAN	Fetuin-B
3	P01857	IGHG1_HUMAN	Immunoglobulin heavy constant gamma 1
4	P01861	IGHG4_HUMAN	Immunoglobulin heavy constant gamma 4
5	P01718	IGLV3–27	Immunoglobulin lambda variable 3–27
6	P20151	KLK2	Kallikrein-2
7	Q8TBK2	SETD6_HUMAN	N-lysine methyltransferase SETD6
8	P18583	SON_HUMAN	Protein SON
9	O95980	RECK_HUMAN	Reversion-inducing cysteine-rich protein with Kazal motifs
10	P02787	TRFE_HUMAN	Serotransferrin
11	P49908	SEPP1_HUMAN	Selenoprotein P
12	P0DOY3	IGLC3_HUMAN	Immunoglobulin lambda constant 3
13	P63241	IF5A1_HUMAN	Eukaryotic translation initiation factor 5A-1
14	P13645	K1C10_HUMAN	Keratin, type I cytoskeletal 10
**Human DNA**			
1	P04264	K2C1_HUMAN	Cluster of Keratin, type II cytoskeletal 1
2	P35527	K1C9_HUMAN	Keratin, type I cytoskeletal 9
3	P13645	K1C10_HUMAN	Keratin, type I cytoskeletal 10

These findings clearly demonstrated that human DNA and eDNA from different bacteria had a distinct influence on the generation of heat-resistant protein fractions. Notably, we did not detect any proteins with decreased heat-resistance following the exposure to eDNA.

**Figure 1:**
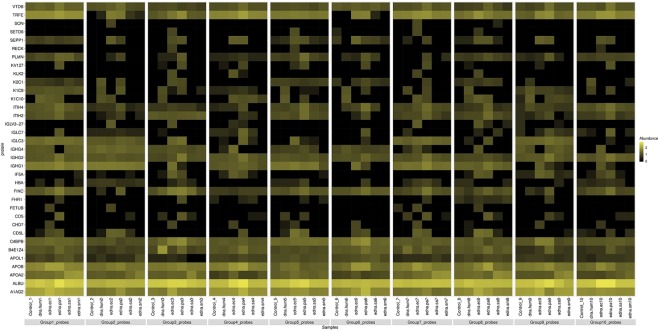
Heatmap of proteins of normal plasma samples that altered their heat resistant characteristics following the treatment with different DNA. The heat map represents the relative effects of DNA from different sources on the proportion of heat-resistant proteins in normal plasma. The colour intensity is a function of protein spectrum counts, with bright yellow and black indicating maximal counts and lack of detection, respectively.

To further analyze the correlation between DNA exposure and acquisition of heat resistance, we constructed a heat map summarizing the impact of different DNAs on the thermal behavior of proteins (Fig. 1).

Plasma exposure to the eDNA of *P. aeruginosa* resulted in the formation of 12 heat-resistant proteins. Notably, only a subset of these proteins, namely K1C10, SEPP1, IGLC3, and IF5A1, also acquired heat resistance after treatment with the DNA of another gram-negative bacteria, *E. coli*. The latter, in turn, changed the heat resistance profile of distinct proteins in the same plasma samples.

Whereas bacterial eDNA induced heat resistance of a broad spectrum of unrelated proteins, plasma exposure to human DNA only affected the thermal behavior of a specific group of proteins, i.e., cytoskeletal keratins.

Since prion domains may be responsible for protein heat resistance, we next employed the prion-prediction PLAAC algorithm to verify the presence of PrDs in proteins exhibiting changes in thermal behavior following DNA treatment.

PrDs were only found in CHD7 and K1C10, which became heat-resistant following the exposure to *E. coli* eDNA, and keratins (K2C1, K1C9, K1C10), which acquired heat resistance upon treatment with both *P. aeruginosa* eDNA and human DNA (Table 3). Notably, the above keratins were the only proteins undergoing thermal behavior alterations following exposure to human DNA.

**Table 3:** Log-likelihood ratio (LLR) score for PrD predictions in plasma proteins that became heat-resistant following DNA treatment.

Protein	LLR Score
CHD7	29.081
K2C1	21.301
K1C9	22.663
K1C10	21.453

We next analyzed the association between DNA-induced changes in protein thermal behavior and human diseases. Surprisingly, the majority of these proteins were previously found to be associated with cancer progression, and some of them are used as a tumor markers (Table 4).

**Table 4:** Association between proteins exhibiting DNA-induced changes in thermal behavior and human diseases

Disease	Proteins	References
Pancreatic cancer	• Serotransferrin • Complement factor H-related protein • Plasma protease C1 inhibitor • Fibronectin • Immunoglobulin lambda constant 7 • C4b-binding protein alpha chain • Selenoprotein P	^45,46,63–71^
Colorectal cancer	APOB SETD6 Reversion-inducing cysteine-rich protein with Kazal motifs (RECK)	^72–74^
Ovarian cancer	Hemoglobin-α Eukaryotic translation initiation factor 5A-1 Fibronectin Inter-α-trypsin inhibitor heavy chain H4 fragment	^75–78^
Breast cancer	Inter-α-trypsin inhibitor heavy chain H4 fragment	^78^
Lung Cancer	ITIH4 Complement Factor H Plasma protease C1 inhibitor Immunoglobulin lambda constant 7 CD5L	^51,79–82^
hairy cell leukemia.	Immunoglobulin kappa variable 1–27	^83^
melanoma	CD5 antigen-like Keratin, type I cytoskeletal 9	^84,85^
Prostatic cancer	Selenoprotein P kallikrein 2 apolipoprotein A-II	^47,86–89^
Bladder cancer	SETD6 Complement factor H-related protein	^90,91^
Thalassemia	HBA	^92^

Intriguingly, some of these cancer-related proteins are also known to be associated with other multifactorial diseases. For example, ITIH4 is associated with schizophrenia and CHD7 is implicated in autism^29–31^.

### Comparison of heat-resistant proteome profile in normal, DNA-treated, and pancreatic cancer plasma

We then examined the changes in protein thermal behavior induced by DNA in normal plasma and compared the resulting pattern with the heat-resistant proteome of patients with pancreatic cancer (Table 5).

**Table 5:** Characteristics of subjects and plasma samples.

Probe	Gender	Age	Tumour Stage	Tumour site	Tumour type
Control 1	F	64	NA	NA	NA
Control 2	F	55	NA	NA	NA
Control 3	M	57	NA	NA	NA
Control 4	M	62	NA	NA	NA
Control 5	M	58	NA	NA	NA
Control 6	F	61	NA	NA	NA
Control 7	F	66	NA	NA	NA
Control 8	M	66	NA	NA	NA
Control 9	M	63	NA	NA	NA
Control 10	F	60	NA	NA	NA
Pancreatic cancer 1	F	63	T3N1M1	Head	Adenocarcinoma
Pancreatic cancer 2	M	57	T3N1M1	Head	Adenocarcinoma
Pancreatic cancer 3	F	56	T3N1M1	Head	Adenocarcinoma
Pancreatic cancer 4	F	69	T3N1M1	Head	Adenocarcinoma
Pancreatic cancer 5	M	61	T3N1M1	Head	Adenocarcinoma
Pancreatic cancer 6	M	52	T3N1M1	Head	Adenocarcinoma
Pancreatic cancer 7	F	59	T2N1M1	Head	Adenocarcinoma
Pancreatic cancer 8	M	72	T3N1M1	Head	Adenocarcinoma
Pancreatic cancer 9	F	64	T2N1M1	Head	Adenocarcinoma
Pancreatic cancer 10	F	71	T3N1M1	Head	Adenocarcinoma

After boiling, the plasma samples of patients with pancreatic cancer were characterized for the presence of heat-resistant proteins. The majority of these proteins were the same that became heat-resistant in normal plasma exposed to DNA treatment. This suggested that DNA exposure was responsible for cancer-related alterations in the thermal behavior of specific proteins.

**Figure 2:**
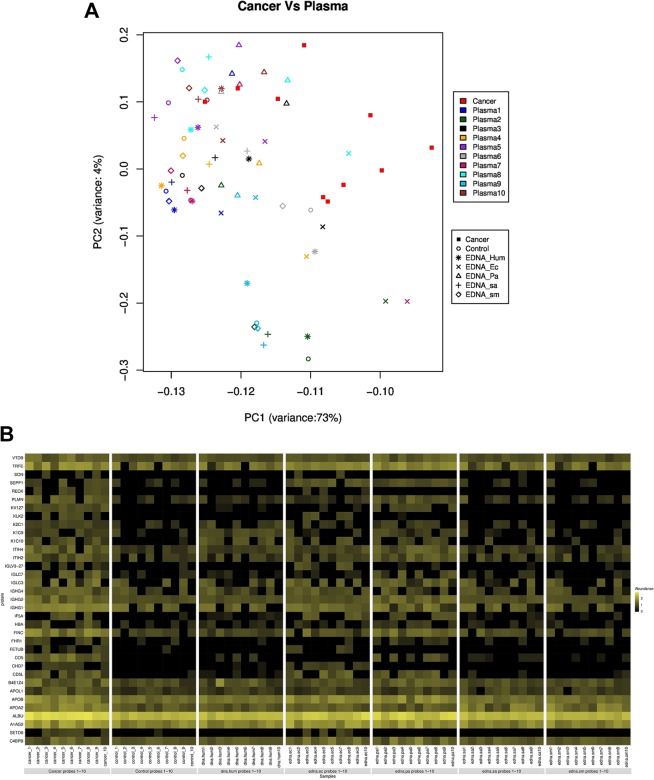
Principal component analysis (PCA) and heat map of proteome data. (**A**) Principal component analysis reflecting the similarities between the heat-resistant proteome of pancreatic cancer plasma and that of plasma from healthy controls following treatment with different DNAs (eby LC/MS). The strongest similarity trend between the plasma of cancer patients and that of healthy subjects after exposure to the eDNA of *P. aeruginosa* are shown. (**B**) Heat map showing the mean spectrum counts of heat-resistant proteins in normal plasma samples following DNA treatment, and in the plasma of patients with pancreatic cancer. Black colour and yellow colours represent low and high spectral counts, respectively.

To further explore the relationship between the heat-resistant proteome of patients with pancreatic cancer and the proteome changes induced by DNA in the plasma of healthy individuals, we analyzed the scaled spectral counts of the identified heat-resistant proteins in both groups by principal component analysis (PCA) (Fig. 2A).

The PCA projection demonstrated that the exposure to bacterial DNA (especially the eDNA of *P. aeruginosa*), induced, in the proteome of normal plasma, changes in thermal behavior.

A heat map based on the highest spectral counts relative to heat-resistant proteins confirmed that treatment of normal plasma with eDNA of *P. aeruginosa* induced a heat-resistant proteome with a higher degree of similarity to that of plasma from cancer patients, compared to that of untreated plasma (Fig. 2B).

## Discussion

This study is the first to demonstrate that bacterial eDNA alters the thermal behavior of specific proteins in human plasma, leading to an increase in the heat-resistant fraction, as well as to the acquisition of heat resistance by proteins that did not exhibit such property prior to DNA exposure.

We discovered that bacterial eDNA or human DNA led to the appearance of different heat-resistant proteins, depending on the DNA source.

Furthermore, we identified a differential effect of eDNA from various gram-positive and gram-negative bacteria on the thermal behavior of plasma proteins. In fact, we surprisingly found that eDNA from different bacteria interacted with distinct plasma proteins (Table 1).

Notably, among the 35 identified proteins with increased heat-resistance following DNA exposure, according to literature data and BindUP tool, only 3 have been previously reported to be able to bind nucleic acids, namely, fibronectin, chromodomain-helicase-DNA-binding protein 7, and SON^32–34^.

Heat resistance was previously described only for complement factor H and fibronectin, whereas the other proteins found to contain heat-resistant fragments in this study were not known to possess this property^35–37^.

Previous studies have shown that one possible mechanism responsible for the acquisition of heat resistance is the formation of β-structures, which confer increased stability to chemical and physical agents^38–42^.

Within this framework, we studied the presence of PrDs in proteins that were found to acquire heat resistance upon DNA exposure and predicted the presence of PrDs only in cytoskeletal and microfibrillar keratins I and II, and in chromodomain-helicase-DNA-binding protein 7^43^. These proteins exhibited a high likelihood ratio (LLR between 21 to 29), and therefore were highly probable to display a prion-like behavior, since the lowest LLR value reported for a known prion-forming protein of budding yeast is ~21.0^44^.

Interestingly, PrD-containing K2C1, K1C9, and K1C10 were the only proteins that were found to acquire heat resistance following treatment with human DNA. In addition, the eDNA from *P. aeruginosa* and *E. coli* induced heat resistance in these PrDs-containing proteins.

The majority of proteins undergoing eDNA-dependent changes in heat resistance identified in the current study did not contain PrDs. This suggested that eDNA caused a PrD-independent induction of heat resistance in these proteins. Therefore, we named proteins undergoing DNA-dependent changes in thermal behavior, in the absence of prion-like structure, “Tetz-proteins”.

Next, we analyzed the association between proteins that acquired DNA-induced heat resistance and human pathologies. According to the literature, many of them were related to a variety of diseases, predominantly cancers. Consistently, some of them are known as tumor biomarkers and participate in tumor progression^45–48^.

Our findings suggested a novel role of bacterial eDNA in disease development, and cancer development in particular, consistent with its reported presence of eDNA in the systemic circulation in association with cancer and other human diseases^20,49^. Indeed, recent studies have shown that patients with non-infectious early-onset cancer display elevated plasma levels of eDNA from bacteria, particularly *Pseudomonas spp*., and *Pannonibacter* spp.^20^. Therefore, for the treatment of plasma samples we used a fixed concentration of bacterial or human DNA, 1 µg/mL, which was selected based on previous studies reporting the presence of similar concentrations of circulating cfDNA in patients with cancer^21,50^.

We next studied the presence and composition of heat-resistant proteins in the plasma of patients with pancreatic cancer. This type of cancer was selected because the majority of proteins that became heat-resistant following bacterial eDNA exposure had been found to be associated with this pathology.

The heat-resistant proteome of cancer patients was compared with that of control plasma, before and after the exposure to eDNA. PCA revealed that, after treatment with different DNAs, the proteome from control plasma acquired non-statistically significant changes in heat resistance that made it more similar to that of plasma samples from cancer patients. We believe that studies on larger sample cohorts may yield statistically significant results. CD5L, EIF5A1, FINC, and SEPP1 particularly attracted our attention as, according to the literature, they are associated with tumorigenesis^46,51–53^. In the present study, heat-resistant fractions of these proteins were identified in pancreatic cancer plasma, but not in normal plasma, and formed only after eDNA treatment, suggesting a role of this conversion in tumorigenesis.

It is tempting to speculate that DNA, including bacterial eDNA, may function as a virulence factor through the interaction with (and the alteration of) Tetz-proteins, including those associated with tumor growth. Therefore, it is possible that under certain conditions, eDNA elevation triggers alterations in plasma proteins that, in turn, may be relevant for tumorigenesis or other pathologies. We also found an effect of eDNA on proteins implicated in neurodegeneration and psychotic disorders. Experiments aimed at the characterization of the pathogenic role of different types of bacterial eDNA are in progress.

## Methods

### Plasma samples

Human plasma samples from 10 healthy donors (age: 55–66 years, 50% females) and 10 patients with clinically diagnosed pancreatic ductal adenocarcinoma (age: 52–72 years, 50% females) were obtained from Bioreclamation IVT (NY, USA), Discovery Life Sciences (Los Osos, CA), Human Microbiology Institute (NY, USA). All patients with pancreatic ductal adenocarcinoma had been diagnosed by histological examination and had not undergone surgical treatment, preoperative chemotherapy or radiotherapy. The basic demographic characteristics of the patients are shown in Table 4. All samples were obtained with prior informed consent at all facilities. Plasma samples were stored at −80 °C until use. This study was approved by the ethics committee of the Human Microbiology Institute (114–40) and all experiments were performed in accordance with relevant guidelines and regulations.

### Extracellular DNA

DNA was extracted from the extracellular matrix of *P. aeruginosa* ATCC 27853, *E. coli* ATCC 25922, *Staphylococcus aureus* ATCC 29213, *Streptococcus mitis* VT-189. All bacterial strains were subcultured from freezer stocks onto Columbia agar plates (Oxoid Ltd., London, England) and incubated at 37 °C for 48 h. To extract the extracellular DNA, bacterial cells were separated from the matrix by centrifugation at 5000 g for 10 min at 4 °C. The supernatant was aspirated and filtered through a 0.2-μm-pore-size cellulose acetate filter (Millipore Corporation, USA). eDNA was extracted by using a DNeasy kit (Qiagen), according to the manufacturer, or by the phenol-chloroform method^54^. Human genomic DNA (0.2 g/L in 10 mM Tris–HCl, 1 mM EDTA, pH 8.0, Cat. No. 11691112001) was purchased from Sigma (Sigma-Aldrich) and consisted of a high molecular weight >50.000 bp genomic DNA isolated from human blood according to the protocol described by Sambrook^55^.

### Plasma exposure to eDNA

DNA was added to plasma samples at the final concentration of 1 µg/mL, incubated at 37 °C for 1 h, and boiled in a water bath at 100 °C for 15 min (by that time all the samples formed clods of coagulated proteins). Samples were cooled at room temperature for 30 min and centrifuged at 5000 g for 10 min at room temperature. The supernatant was aspirated and filtered through a 0.2-μm pore size cellulose acetate filter (Millipore Corporation, USA).

### Protein identification by LS-MS

The filtered protein-containing supernatant was diluted in a final volume of 100 µL using 100 mM ammonium bicarbonate, pH 8, and quantified using a Nanodrop OneC Spectrophotometer (Thermo Fisher Scientific). Cysteine residues were reduced using 5 mM dithiothreitol at room temperature for 1.5 h and alkylated with 10 mM iodoacetamide at room temperature for 45 min in the dark. Proteins were then digested using modified trypsin (Promega, P/N V5113) at a 1:20 (w/w) enzyme:protein ratio for 16 h at 22 °C. After digestion, peptides were acidified to pH 3 with formic acid and desalted using Pierce Peptide Desalting Spin Columns (P/N 89852), according to the manufacturer’s protocol. Eluted, desalted peptides were dried down to completion using a Labconco speedvac concentrator, resuspended in 0.1% formic acid and quantified again using a Nanodrop OneC Spectrophotometer^56^. For sample injection and mass analysis, peptides were diluted to a final concentration of 500 ng/µL using 0.1% formic acid in water to provide a total injection amount of 500 ng in a 1 µL of sample loop. Peptides were separated and their mass analyzed using a Dionex UltiMate 3000 RSLCnano ultra-high performance liquid chromatograph (UPLC) coupled to a Thermo Scientific Q Exactive HF hybrid quadrupole-orbitrap mass spectrometer (MS). A 1.5 hr reversed-phase UPLC method was used to separate peptides using a nanoEASE m/z peptide BEH C18 analytical column (Waters, P/N 186008795). The MS method included top 15 data-dependent acquisition for interrogation of peptides by MS/MS using HCD fragmentation. All raw data were searched against the human Uniprot protein database (UP000005640, accessed Apr 22, 2017) using the Andromeda search algorithm within the MaxQuant suite (v 1.6.0.1)^57,58^. The search results were filtered to a 1% FPR and visualized using Scaffold (v4, Proteome Software).

A cut-off of at least 5 spectral counts per probe was applied for protein selection^59–61^.

The obtained data were used to generate a heatmap. The abundance values were log converted (zero values were replaced with infinitely small number “1”) and plotted with R-statistical computing (https://www.r-project.org/), using the “levelplot” package. The color key indicates a range between the lowest (black) and the highest (yellow) values.

Principal components analysis was performed using the prcomp function with default parameters (zero values were replaced with 1) of the R software (https://www.r-project.org/).

### Identification of prion-like domains (PrDs) in proteins

The presence of prion-like domains in the proteins was assessed using the PLAAC prion prediction algorithm, which establishes the prionogenic nature on the basis of the asparagine (Q) and glutamine (N) content, using the hidden Markov model (HMM)^43,62^. The output probabilities for the PrD states in PLAAC were estimated based on the amino acid frequencies in the PrDs of *Saccharomyces cerevisiae*. Here, we used Alpha = 0.0, representing species-independent scanning, to identify the PrDs.

## Data Availability

The other sequencing datasets generated and/or analyzed during the current study are available from the corresponding author on reasonable request.
